# Seasonal shifts in feeding patterns: Individual and population realized specialization in a high Arctic fish

**DOI:** 10.1002/ece3.5615

**Published:** 2019-09-21

**Authors:** Marine Cusa, Jørgen Berge, Øystein Varpe

**Affiliations:** ^1^ Department of Arctic Biology The University Centre in Svalbard Longyearbyen Norway; ^2^ Department of Arctic and Marine Biology UiT ‐ The Arctic University of Norway Tromsø Norway; ^3^ Akvaplan‐niva Fram Centre Tromsø Norway

**Keywords:** Arctic cod, *Boreogadus saida*, diet, niche width, polar cod, predator–prey interactions

## Abstract

Species with a broad and flexible diet may be at an advantage in a rapidly changing environment such as in today's Arctic ecosystems. Polar cod (*Boreogadus saida*), an abundant and ecologically important circumpolar Arctic fish, is often described as a zooplankton generalist feeder, which suggests that it may cope successfully with changes in prey composition. This description is justified based on the relatively broad diet of polar cod across sites and seasons. In this case study, we used polar cod dietary data from fall and winter and from two distinct environments, dominated either by Arctic or Atlantic water masses in Svalbard. Our results point to the importance of time and space when drawing conclusions on dietary plasticity and degree of specialization. Polar cod diet differed significantly between fall and the winter and between Arctic and Atlantic domains. Polar cod from Arctic domains displayed a strong realized population specialization on *Themisto libellula* in fall, and the larger dietary niche width observed in the winter was the product of realized individual specialization, with increased feeding on fish prey. Overall, we did not observe a generalized feeding behavior. If dietary niche width is to inform conservation management, we argue it must be recognized that populations from a single species may adopt seasonally contrasting degrees of dietary specialization and that these populations may differ in their vulnerability to climate‐induced changes in prey community composition.

## Graphical abstract

By taking an alternative approach to dietary analysis, this article challenges the commonly held assumption that polar cod is a generalist predator and argues that it can display strong temporal and geographical dietary specialization.

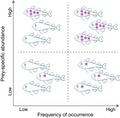

## 1 INTRODUCTION

Generalist feeders display large dietary niche width comprising of high prey taxa diversity, whereas specialists display narrow dietary niche width composed of few prey taxa (Gerking, 1994). To predict the vulnerability of a species to environmental disturbances, it is essential to measure its niche width and degree of specialization. Ecological generalists will, for example, often outperform specialists in a degraded environment ultimately leading to a reduction in species diversity (Clavel, Julliard, & Devictor, 2011; Devictor, Julliard, & Jiguet, 2008). Yet, set definitions and systematic quantification measures are often missing when assessing attributes pertaining to degrees of ecological specialization (Ferry‐Graham, Bolnick, & Wainwright, 2002), which can lead to incomparable studies (Ferry‐Graham et al., 2002) and ineffective management measures (Devictor et al., 2010). Indeed, the concept of ecological specialization is flexible yet frequently treated as a rigid species attribute.

To describe dietary specialization, we can use ecological levels other than species, such as individual, population, or community (Devictor et al., 2010; Fox & Morrow, 1981). For instance, populations from a generalist species can be composed of generalist individuals displaying a high within‐individual variation in resource use or of specialist individuals displaying a high between‐individual variation in resource use (Amundsen, Gabler, & Staldvik, 1996; Bearhop, Adams, Waldron, Fuller, & MacLeod, 2004). Additionally, the degree of specialization need not be static in time and space as a species realized dietary niche width can vary seasonally and geographically (Devictor et al., 2010; Ferry‐Graham et al., 2002). Thus, *fundamental specialization* describes a degree of specialization that is an inherent species trait, and *realized specialization* describes a degree of specialization that is flexible and dependent on local conditions.

Polar cod (*Boreogadus saida*) is ubiquitous both in open water and below the pack‐ice (Gradinger & Bluhm, 2004; Lønne & Gulliksen, 1989) and is the most abundant species in epipelagic zones at high latitudes (Benoit, Simard, & Fortier, 2008; Fortier et al., 2015). Polar cod plays an important role in linking trophic levels and is responsible for a large transfer of energy to piscivorous organisms (Hop & Gjøsæter, 2013; Wassmann et al., 2006; Welch et al., 1992). The presence of polar cod in both the Atlantic and Arctic domains in Svalbard suggests that this species has important adaptive capacities and can tolerate a wide range of temperatures and salinity (Falk‐Petersen, Frivoll, Gulliksen, & Haug, 1986; Nahrgang et al., 2014; Renaud et al., 2012) though it was suggested that polar cod's growth and fecundity is reduced in Atlantic domains (Nahrgang et al., 2014). Based on pan‐arctic meta‐analysis, it was established that polar cod is a zooplankton generalist predator (Mueter, Nahrgang, Nelson, & Berge, 2016; Renaud et al., 2012). Polar cod have also been described as opportunist feeders (Ajiad & Gjøsæter, 1990; Majewski et al., 2016; Nakano et al., 2016) and more rarely as specialist feeders (Cui, Grebmeier, & Cooper, 2012). This variability partly stems from evaluating diet at varying spatial and temporal scales and from inconsistencies regarding the choice of a fundamental or realized approach to describing degrees of specialization. Furthermore, the difficulties and expenses associated with sampling during the polar night have greatly hampered seasonal comparisons and this study offers rare insights into seasonal variation of ecological processes in the high Arctic.

Here, we studied polar cod stomach content to evaluate and contrast the (a) seasonal and geographical variations in dietary niche width, (b) variation in ingested prey taxa between fall and winter from Atlantic and Arctic domains, and (c) degree of realized individual and population specialization in fall and winter from Arctic domain fjords and an Atlantic domain fjord using a metric of specialization developed by Amundsen et al. (1996). More generally, we aim to reveal the importance of acknowledging organizational levels (individual, population, and species) when drawing conclusions on dietary specialization.

## 2 MATERIALS AND METHODS

### 2.1 Field sampling

**Figure 1:**
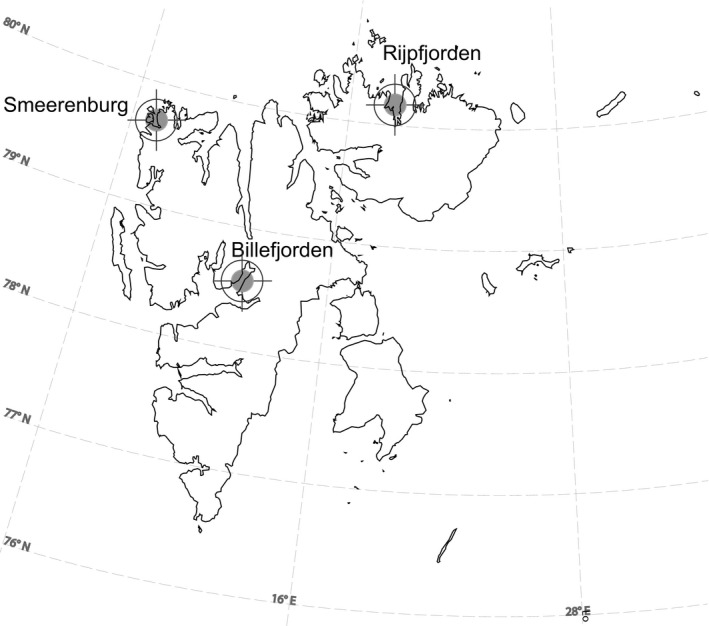
Map of Svalbard illustrating the three sampling locations; Rijpfjorden and Billefjorden being typically Arctic domain fjords and Smeerenburg being typically an Atlantic domain fjord

Polar cod were collected in three sites from the Svalbard archipelago. Billefjorden and Rijpfjorden are fjords displaying Arctic characteristics and dominated by Arctic species (Bandara et al., 2016; Nahrgang et al., 2014), whereas the sound of Smeerenburg is heavily influenced by inflow of warmer and more saline Atlantic water (Figure 1). The fish were caught using a Campelen 1800 bottom trawl with a 22 mm cod‐end mesh size aboard the R/V Helmer Hanssen. Trawls were conducted in September and October 2014, January 2015, September 2015, and January 2016, with 11 trawls in total and at depths ranging from 158 to 267 m.

Polar cod were weighed, measured, and divided into three size categories, <10, 10–15, and >15 cm, based on Falk‐Petersen et al. (1986). Only individuals over 10 cm were kept for the analysis as they are likely to be mature individuals and be less limited in their diet choice due to gape size. The dissected stomachs were examined with a dissecting microscope, and prey taxa were identified to the lowest possible taxonomic level. Individuals of each prey taxa were counted and weighed. Some taxonomic levels were grouped into broader dietary categories for the analyses.

### 2.2 Qualitative diet analysis

**Figure 2:**
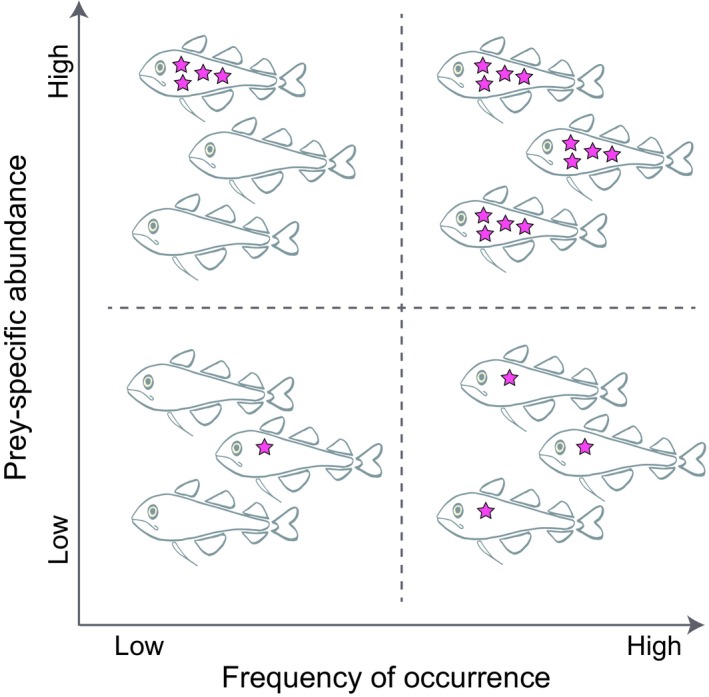
Schematic illustration adapted from the feeding strategy diagram of Amundsen et al. (1996). The original diagram that operates on a continuum was here simplified into four categories representative of four extreme circumstances at each corner of the diagram. Thus, the top left corner represents a situation with 100% prey‐specific abundance (P*i*) in a single fish highlighting an extremely high between‐individual component (also called between‐phenotype component by Amundsen et al. (1996)), the top right corner represents a situation with the highest FO and 100% P*i* illustrating an extremely high population specialization, the bottom left corner represents a situation with low FO and low %P*i*, and the bottom right corner a situation with high FO and low %P*i* illustrating a high within‐individual component (also called within‐phenotype component by Amundsen et al. (1996)) and a population generalization

In order to observe whether polar cod displayed seasonal variations in diet, we first performed a nonmetric multidimensional scaling (nMDS) analysis. Polar cod level of specialization was then directly assessed using the percent prey‐specific abundance (%P*i*) versus frequency of occurrence (FO) diagram described by Amundsen et al. (1996) (Figure 2). FO and %P*i* were measured using the following equations:

$$\text{FO}=\left(N_{i}/N\right)$$

$$\%\;Pi=\left(\sum S_{i}/\sum S_{t_{i}}\right)\times 100$$

in which *N_i_* is the number of stomachs with a given prey *i* in their stomach, *N* is the total number of stomachs excluding empty stomachs, *S_i_* is the total weight of prey *i* from all stomachs, and in which $S_{t_{i}}$ is the total prey weight of all stomachs containing prey *i*. Empty stomachs were excluded from the calculations along with unidentified material which would bias the results. Diagrams were generated separately for the three sites and the two seasons, and all individuals with prey in their stomach (*n* = 286) were retained for the analysis. Dietary niche width was calculated with the Shannon–Wiener (H′) diversity index (Spellerberg & Fedor, 2003) and Simpson's (D) diversity index (Simpson, 1949).

### 2.3 Statistical diet analysis

Because of the nonindependent nature of our data, we first performed a multivariate analysis. Since our data did not meet the assumptions of a multivariate analysis of variance (MANOVA), we used a multivariate permutation analysis of variance (PERMANOVA; Anderson, 2001) to identify potential differences in diet composition between seasons and domains. The classical *F* test using both an analysis of variance (ANOVA) and a permutation test confirmed that our data met the assumptions of a PERMANOVA. For each sample site, prey data were normalized by dividing each measure of abundance by the total number of individual polar cod to avoid over‐representation of a prey for a given site when a higher number of polar cod from that site was analyzed. For the statistical analysis, all fish prey data (*B. saida*, *Leptoclinus* sp., *Sebastes* sp., and nonidentified fish) were pooled under the category Teleostei.

For statistical inference on the qualitative specialization metrics from the Amundsen et al. (1996) diagrams, we selected several preys of interest and tested their relative gravimetric abundance between sites and seasons using the Kruskal–Wallis test (*α* ≤ .005). The mean relative abundance of a given prey *x* ($\bar{x}A$) is a good approximation of the frequency of occurrence (FO) of that prey as it accounts for all stomachs including those that do not contain prey *x* (but excluding empty stomachs). Therefore, high mean relative abundance is likely to reflect high frequency of occurrence (FO) of prey *x*. Mean relative abundance as a proxy for FO was calculated as:

$$\left(\bar{x}A\right)=\frac{1}{n}\times \sum_{j=1}^{n}{\left(N_{x}/N_{T}\right)}_{j}$$

in which *n* is the total number of stomachs, *N_x_* is the total amount of prey *x* for fish *j*, and *N*_T_ is the total amount of prey for fish *j*. For consistency with the Amundsen et al. (1996) diagram, the amounts are measured in grams, but the method could also apply to numerical quantities.

The mean relative abundance of prey *x* *in prey‐specific stomachs* ($\bar{x}A_{p}$) is a good approximation of prey‐specific abundance (P*i*) as it focuses strictly on stomachs containing prey *x*. Therefore, high mean relative abundance of prey *x* in prey‐specific stomachs is likely to reflect high prey‐specific abundance (P*i*). Mean relative abundance in prey‐specific stomachs as a proxy for P*i* was calculated as:

$$\left(\bar{x}A_{p}\right)=\frac{1}{n_{p}}\times \sum_{j=1}^{n_{p}}{\left(N_{x}/N_{T}\right)}_{j}$$

in which *n*_p_ is the total number of stomachs containing prey *x*.

High mean relative abundance ($\bar{x}A$) and high mean relative abundance *in prey‐specific stomachs* ($\bar{x}A_{p}$) are analogous to general specialization, whereas low mean relative abundance ($\bar{x}A$) but high mean relative abundance *in prey‐specific stomachs* ($\bar{x}A_{p}$) is analogous to individual specialization. The Kruskal–Wallis analysis was conducted on the most abundant prey types (as per gravimetric measures): *Themisto libellula*, Teleostei, and Euphausidae. These results combined with a visual interpretation of Amundsen et al. (1996) diagrams were used to evaluate the difference in the degree of specialization between sites and seasons. All statistical analyses were performed using the R Core Team (2014) software.

## 3 RESULTS

### 3.1 Effects of season and domains on diet composition

**Figure 3:**
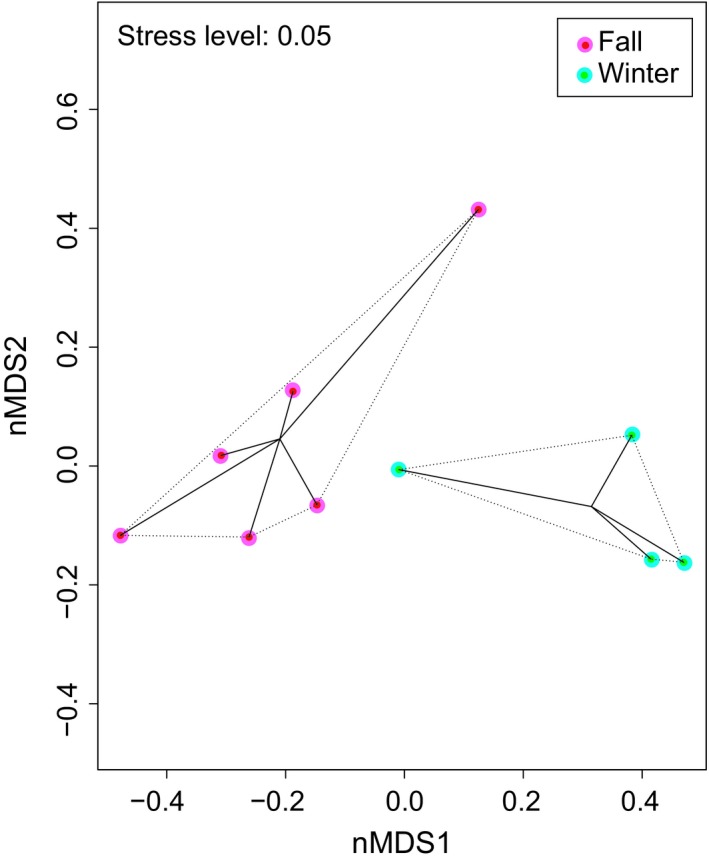
Nonmetric multidimensional scaling (nMDS) ordination of polar cod (*Boreogadus saida*) stomach content from Svalbard fjords during two different seasons: fall and winter

We retained 309 stomachs for the diet analysis from a total of eleven trawl samples across two domains (Arctic and Atlantic) and seasons (Fall and Winter). The nMDS highlighted a seasonal effect in dietary composition for all populations (Figure 3). The PERMANOVA indicated that polar cod diet differed significantly between fall and winter (*F*_1,6_ = 7.3167; *p* = .002), and between Arctic and Atlantic domains (*F*_1,6_ = 2.69; *p* = .042); 40% of the total variance was explained by the Season factor (*r* ^2^ = .401), whereas 15% of the total variance was explained by the Domain factor (*r* ^2^ = .147). A SIMPER analysis indicated that *T. libellula* and Teleostei contributed to at least 70% of the observed difference between groups. Characterization of the seasonal diets follows in the analysis on degree of specialization.

### 3.2 Degree of specialization

**Figure 4:**
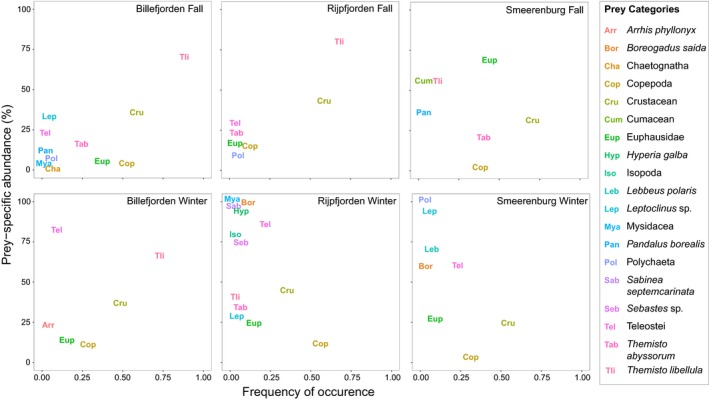
Percent prey‐specific abundance (%P*i*) versus frequency of occurrence (FO) for polar cod from three Svalbard fjords during two different seasons: fall and winter

**Figure 5:**
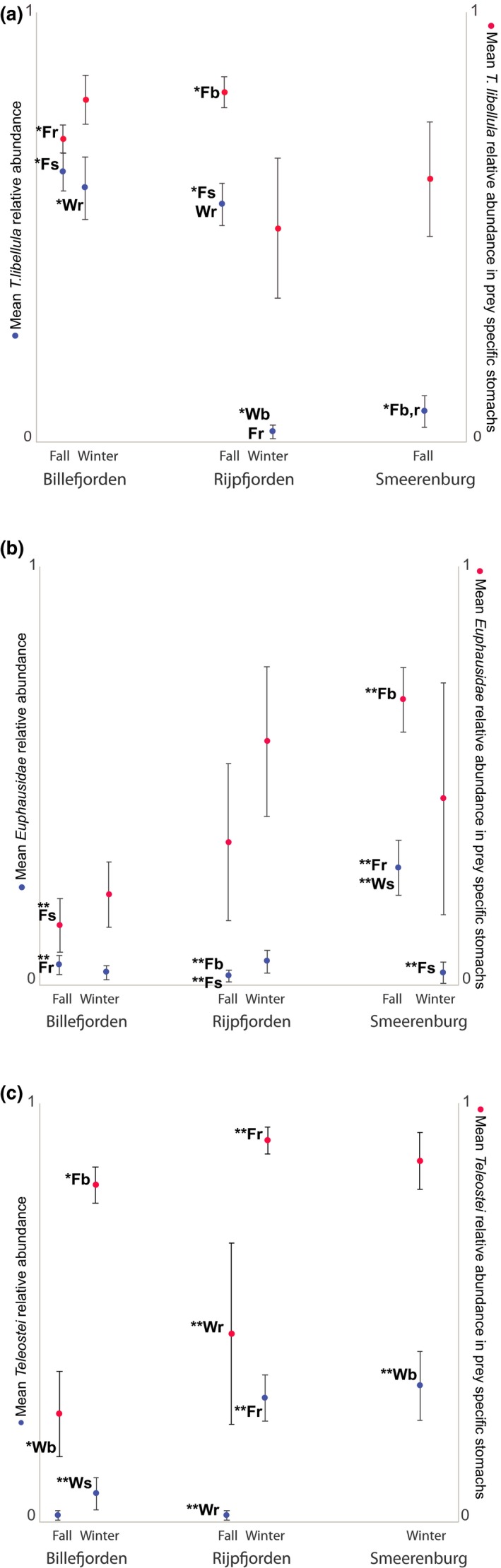
Mean value and error bars for relative (a) *T. libellula*, (b) Euphausidae, and (c) Teleostei abundance for polar cod from Billefjorden, Rijpfjorden, and Smeerenburg. The mean relative abundance of prey *x* in prey‐specific stomachs focuses strictly on stomachs containing prey *x*. Letters above mean value points indicate statistically significant difference from indicated site/season (F = Fall, W = Winter, s = Smeerenburg, r = Rijpfjorden, b = Billefjorden), *p < .005 and **p  < .001 (Kruskal–Wallis test)

A total of 286 polar cod were included in the analysis of degree of specialization. Population specialization on *T. libellula* was observed for both Arctic sites (Billefjorden and Rijpfjorden) in fall. This was illustrated both by the Amundsen et al. (1996) diagrams (Figure 4) and by Figure 5a (high mean relative abundance [$\bar{x}A$] and high mean relative abundance for prey‐specific stomachs [$\bar{x}A_{p}$]). In the Atlantic site of Smeerenburg, no such specialization was present, and Billefjorden and Rijpfjorden both exhibited a significantly different mean relative abundance of *T. libellula* from Smeerenburg in fall suggesting a lower frequency of occurrence of *T. libellula* in the Atlantic site (Figure 5a). In Smeerenburg, euphausiids prey had a relatively high frequency of occurrence and prey‐specific abundance (Figures 4 and 5b) approaching a population specialization on this taxa.

**Figure 6:**
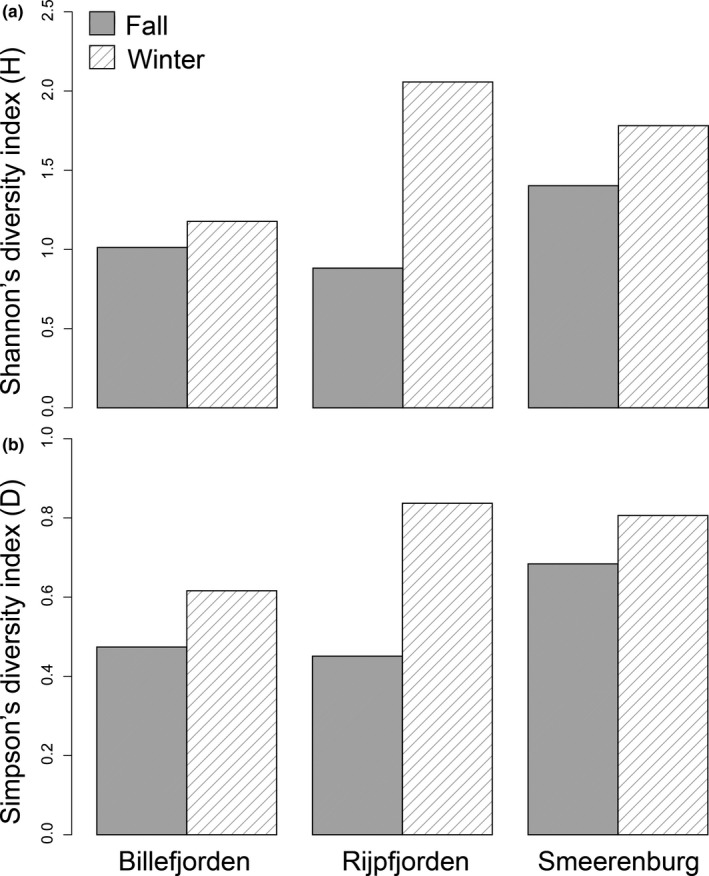
(a) Dietary niche width calculated with Shannon–Wiener (H′) and (b) Simpson's (D) diversity index separated for each site and season

In the winter, only the Arctic fjord of Billefjorden still displayed population specialization on *T. libellula*, and individual specialization on fish prey was present at all sites. Though both Billefjorden and Rijpfjorden are Arctic domain fjords, the winter dietary niche width was wider in Rijpfjorden than Billefjorden (Figure 6a) with the frequency of occurrence of ingested *T. libellula* being much lower in Rijpfjorden. In the Atlantic site of Smeerenburg, fish prey had the highest frequency of occurrence and the highest prey‐specific abundance (Figures 4 and 5c). A shift from a realized population specialization in fall to a realized individual specialization in the winter was visible when examining each site separately (Figure 4). Overall, we did not observe a generalized feeding behavior (or high within‐individual component).

### 3.3 Dietary niche width and prey diversity

In concordance with population specialization in Arctic sites in fall, the Shannon–Wiener index highlighted an increase in dietary niche width in the winter in each site and a greater dietary niche width in the Atlantic site in fall (Figure 6a). These trends are also displayed by the Simpson's index, which indicated a higher ingested prey diversity in the winter for all sites, and a higher ingested prey diversity in the Atlantic site in fall (Figure 6b).

## 4 DISCUSSION

### 4.1 Spatial and temporal variations in polar cod diet and degree of specialization

Polar cod is commonly depicted as an essential element of the Arctic marine food web linking primary consumers to higher trophic levels (Bradstreet et al., 1986; Melnikov & Chernova, 2013; Rice, 1995). Our study indicates that adult polar cod populations from Svalbard also act as tertiary consumers with a diet primarily composed of secondary consumer amphipod in fall and fish prey in the winter.

We noted a strong population specialization on *T. libellula* in Arctic domains in fall, which corresponds to prior diet observations even in the presence of other prey types such as euphausiids and copepods (Dalpadado et al., 2016). A diet primarily composed of the hyperiid amphipod *T. libellula* in Arctic domains in fall had previously been reported in a study comparing the diet and reproduction of polar cod between Atlantic and Arctic domains in Svalbard (Nahrgang et al., 2014). Likewise, *T. libellula* is frequently observed as a prey item in the North American Arctic, but important interannual variations exist (Buckley & Whitehouse, 2017). Studies from the North American Arctic report a variety of ingested amphipod species with the predominance of set families or suborders depending on the region, essentially the Hyperiidea amphipod species *T. libellula* and *Themisto abyssorum* (Majewski et al., 2016), the Senticaudata amphipod, *Apherusa glacialis* (Walkusz, Majewski, & Reist, 2013), and Ampeliscidae amphipods, a family of the suborder Gammaridea (Cui et al., 2012). Our study highlights the ingestion of *T. abyssorum* in the Atlantic site of Smeerenburg in line with evidence supporting the presence of this amphipod in Atlantic domains (Ormańczyk, Głuchowska, Olszewska, & Kwasniewski, 2017).

We observed a shift in diet from autumn to winter with fish prey becoming relatively important. Recent publications have shown how environmental factors such as temperature can affect the nutritional value of a given prey (Rosenblatt & Schmitz, 2016), which could explain predator's seasonal diet shift. The larger niche width in the winter is seemingly analogous to a generalist diet, but the diagrams indicate a strong individual specialization and between‐individual variation in resource use. Although polar cod occasionally ingest juvenile teleosts (Buckley & Whitehouse, 2017; Craig, Griffiths, Haldorson, & McElderry, 1982; Cui et al., 2012; Majewski et al., 2016; Rand et al., 2013), these results are surprising as the contribution of this prey to polar cod diet is often deemed negligible (Buckley & Whitehouse, 2017; Rand et al., 2013). Other prey species occasionally dominate polar cod diet such as mysiids (Craig et al., 1982) or euphausiids (Rand et al., 2013), and our results indicate that euphausiids ingestion seems particularly enhanced in Svalbard's Atlantic domain sites where euphausiids are dominating and where the abundance of *T. libellula* varies widely interannually and decreases with stronger inflow of Atlantic water (Dalpadado et al., 2016).

Altering prey availability as a result of seasonality and habitat heterogeneity, and abiotic factors such as seasonal change in light regime are suspected to explain these changes in ingested prey species compositions. These observations have led many to conclude that polar cod are opportunistic feeders (Ajiad & Gjøsæter, 1990; Bradstreet et al., 1986; Buckley & Whitehouse, 2017; Christiansen, Hop, Nilssen, & Joensen, 2012; Lowry & Frost, 1981) and zooplankton generalists (Renaud et al., 2012) and will feed on which ever prey is available and most abundant at a given moment. Our study confirms that polar cod display a generalist diet at the species level with a diverse set of invertebrates as well as fish prey, but it also highlights a strong realized specialization that varies seasonally and geographically.

### 4.2 The biotic and abiotic mechanisms for a realized specialization

Polar cod have the ability to forage on a wide diversity of prey yet when compared to other co‐occurring fish species, their diet often suggests a population specialization that does not appear to reflect the diversity of available prey (Cui et al., 2012; Dalpadado et al., 2016). Optimal diet theory (ODT) predicts that an increase in prey abundance and diversity should lead generalist predators to discriminate between preys and to specialize on higher value prey types (Sih & Christensen, 2001). Arctic fjords generally exhibit a diversity of potential prey such as *T. libellula*, *T. abyssorum*, euphausiids, and large calanoid copepods (Bandara et al., 2016; Cusa, 2016; Gluchowska et al., 2016; Ormańczyk et al., 2017), and the observed specialization on *T. libellula* could be the result of a seasonal behavioral specialization by a discriminating generalist. The predominance of *T. libellula* in the diet could also reflect a facility to catch this prey over other prey for a variety of reasons including handling ease, prey's color and visibility, aggregation and swimming behavior, and avoidance behavior.

If polar cod demonstrate a behavioral specialization in Arctic fjords in fall, this behavior is likely to be altered by seasonality, not just as a result of the declining abundance of a preferred prey, but also because of seasonal light regimes and the dark winter conditions encountered at high latitudes during the polar night. A decrease in light reduces the prey detection distance of visual predators (Aksnes & Utne, 1997; Langbehn & Varpe, 2017) possibly leading to a more generalized diet during the winter months. A change in foraging tactics shifting from pelagic to benthic could further explain the observed change in diet (Jørgensen & Jobling, 1990). Interestingly, the winter diet of polar cod remains quite specialized on *T. libellula* in Billefjorden. Polar cod's ability to adjust to a low‐light regime by changing the optical properties of its lens to a rod‐dominated retina while retaining multifocal vision (Jönsson, Varpe, Kozłowski, Berge, & Kröger, 2014) combined with more light in this lower latitude fjord (Berge et al., 2015) may permit them to continue foraging on *T. libellula*.

In 2017, potential fish prey species were all more abundant in summer than in winter (Geoffroy et al., 2019), yet the rather consistent individual specialization on these prey predominantly in winter suggests that other mechanisms than prey availability may be at play, such as the impaired visual ability of fish prey to escape their predators during the winter months, though polar cod might be equally impaired visually.

### 4.3 Limitations

One of the most important limitations of this study is the lack of prey community composition systematically sampled in concurrence with polar cod stomach samples. Methot Isaac Kidd (MIK) sampling was performed both in Rijpfjorden in fall and in winter for an adjacent project but the samples were analyzed using different methods and levels of expertise. We therefore prefer to discuss our results by referring to past studies and other manuscripts, which have investigated the community composition and abundance of zooplankton in a range of fjords in Svalbard allowing us to make well‐informed inferences (Bandara et al., 2016; Błachowiak‐Samołyk et al., 2017; Cusa, 2016; Dalpadado et al., 2016; Geoffroy et al., 2019; Kraft, Berge, Varpe, & Falk‐Petersen, 2013). Furthermore, bioenergetic measurements of the potential prey would help elucidate whether, all else being equal, there is an advantage to select for a given prey over other available prey (But see Mayzaud & Boutoute, 2015). Such information should be combined with polar cod physiological attributes and its seasonally varying energy requirements. Finally, a stable isotope analysis would nicely complete this seasonal study by bringing further insight on the seasonal and spatial component of the observed specializations. Stable isotope analysis of polar cod from Svalbard has previously been conducted (McGovern, Berge, Szymczycha, Węsławski, & Renaud, 2018; Renaud et al., 2012), but it is difficult to draw conclusions based on these analyses as they diverge from our samples both spatially and temporally.

### 4.4 Implications, outlook, and conclusion

Genetic variation between polar cod populations (Madsen, Nelson, Fevolden, Christiansen, & Præbel, 2016), their possible isolation, as well as a lack of understanding on migratory and reproductive patterns, calls for a narrower geographical scale description of feeding strategies. It also asks for careful consideration when implying that putative populations of polar cod may respond homogeneously to changes in prey composition. Populations that exhibit strong genetic structure could display unique population‐specific spatial and temporal specialization (Siwertsson et al., 2013). If, on the contrary, polar cod populations experience high gene flow, combining dietary data with physiological measurements might reveal essential information regarding the importance of seasonal prey selectivity and its impact on polar cod growth and fecundity. Studies have demonstrated that environmental factors can affect metabolic rate, thereby changing a predator's energetic requirements and its feeding strategy (Rowe, Figueira, Raubenheimer, Solon‐Biet, & Machovsky‐Capuska, 2018). Considering that polar cod population specialization occurs in fall, at a time period when energetic requirements are particularly high (prewinter and prereproduction, Nahrgang et al., 2014), understanding whether polar cod populations should be treated as distinct units or not seems particularly relevant.

The Arctic environment is changing rapidly, altering trophic interactions, selection pressures, and community composition (Edwards & Richardson, 2004; Hoegh‐Guldberg & Bruno, 2010; Langbehn & Varpe, 2017; Rice, 1995; Varpe, Daase, & Kristiansen, 2013; Zerba & Collins, 1992); and species response to changes induced by climate change will partly depend on their phenotypic plasticity (Munday, Warner, Monro, Pandolfi, & Marshall, 2013; Sih, Ferrari, & Harris, 2011). Studies have previously reported the selective behavior of polar cod on specific prey types (Cui et al., 2012; Hop & Gjøsæter, 2013), and we therefore suggest that polar cod specialization on *T. libellula* in Arctic domains in fall is a factor of both (a) an increased availability of *T. libellula*, and (b) a preference for *T. libellula* as a prey. Furthermore, we suggest that the near‐absence of *T. libellula* in the diet of polar cod in some sites during the winter and an increase in dietary niche width including individual specialization on fish prey likely reflects either or both (a) the low abundance of this Arctic amphipod, and (b) fish's reduced ability to rely on visual cues for prey search and predator avoidance.

## CONFLICT OF INTEREST

The authors declare that they have no conflict of interest.

## AUTHOR CONTRIBUTIONS

The article is based on the Master of Science project of MC. MC designed the study, collected and analyzed the data, and wrote the manuscript. JB advised on design, organized cruise time, and gave editorial advice on the manuscript. ØV advised throughout the study, participated in the design and writing, and provided support in data collection, analysis, and interpretation.

## HUMAN AND ANIMAL RIGHTS

All applicable institutional and/or national guidelines for the care and use of animals were followed. Sampling and processing of polar cod was performed in compliance with the Norwegian animal welfare authorities.

## Data Availability

Data supporting this article are available on Dryad: https://doi.org/10.5061/dryad.6472vq6
